# The impact of virtual synchronous compensator on the transient synchronous stability of renewable energy

**DOI:** 10.1038/s41598-026-34998-5

**Published:** 2026-02-09

**Authors:** Fan Sun, Yongping Chen, Weiqing Wang

**Affiliations:** 1 College of Electrical Engineering, Xinjiang University, Urumqi, 830046 China; 2 Bazhou Electric Power Company, Grid State Xinjiang Electric Power Co.LTD, Kuerle, China

**Keywords:** Virtual synchronous compensator, Renewable energy grid-connected converter, Current-Limiting control strategy, Transient synchronous stability, Energy science and technology, Engineering

## Abstract

The virtual synchronous compensator (VSCOM) integrates virtual synchronous machine control within a static var generator (SVG), providing active voltage support and improving the adaptability of SVGs to weak grid conditions. However, the interaction between VSCOM, which adopts grid-forming control, and renewable energy grid-connected converters (REGC) based on grid-following control introduces complex transient stability characteristics. This study investigates the effect of VSCOM on the transient synchronous stability of REGC under large grid disturbances. First, a constant voltage current-limiting control strategy for VSCOM is proposed based on its operational characteristics. A mathematical model is then established to assess the enhancement of the static stability limit of REGC by VSCOM. Subsequently, a transient model of the coupled VSCOM-REGC system is developed, considering the short-circuit ratio (SCR), control parameters, and reactive power capacity, to clarify the mechanism by which VSCOM affects the transient synchronous stability of REGC. Finally, an electromagnetic transient simulation model is built using MATLAB/Simulink to verify the theoretical analysis.

## Introduction

With the rapid development of renewable energy in the new-type power system construction, large-scale wind and photovoltaic generation are increasingly integrated into the grid using grid-following (GFL) controlled inverters^1,2^. GFL inverters synchronize with the grid via Phase-Locked Loop (PLL) control, but lack the active voltage support capability inherent to synchronous generators. As power systems shift from synchronous machine dominance to inverter-based control, grid strength gradually declines, creating significant challenges for the stability of power systems with high shares of renewable energy generation^3–6^.

To address voltage deviations at the point of connection (POC) in renewable energy plants—caused by the fluctuating output of renewable sources and long-distance power transmission^7,8^—reactive power compensators are commonly installed at the POC in engineering practice to stabilize voltage^9^. The most widely used reactive power compensators in renewable energy plants are static var compensators (SVCs) and static var generators (SVGs)^10,11^.

SVCs provide reactive power compensation by continuously adjusting the equivalent grid admittance through thyristor-controlled capacitors and reactors^12^. SVGs use PLL control to synchronize with the grid and directly regulate reactive current to provide reactive power compensation for renewable energy plants^13,14^. However, the essential purpose of reactive power compensation is to deliver effective voltage support at grid nodes. Given that SVCs and SVGs act as variable admittance devices and current sources—and considering the poor stability of PLL-based control under weak grid conditions—these technologies face significant limitations in providing voltage support in such scenarios^15,16^.

To overcome these challenges, researchers have proposed Virtual Synchronous Compensator (VSCOM) control technology, which integrates Grid-Forming (GFM) control into existing SVG devices^17–19^. VSCOMs can autonomously generate voltage phase angles, establish a constant AC voltage at the POC, and actively regulate plant voltage as voltage sources. This enables active voltage support for the grid and addresses the shortage of voltage sources in new-type power systems^20,21^. Nevertheless, existing research on VSCOMs has mainly focused on synchronous control loops. Reference^22^ utilizes current detection and incorporates a synchronization loop compensation term to adaptively adjust the control mode, enabling GFM to achieve transient stability under large disturbances. However, as the VSCOM operates as a GFM reactive power compensator, it lacks the additional energy source required by this method for active power loop correction and compensation, making the method difficult to apply to VSCOM. Therefore, the method in^22^ is difficult to apply to VSCOM. Furthermore, studies on current-limiting control strategies that maintain the voltage source characteristics of VSCOMs during grid voltage sags remain incomplete^23,24^.

Moreover, during severe grid disturbances such as short-circuit faults, renewable energy grid-connected converters (REGCs) operating under weak grid conditions with PLL-based control may experience transient synchronous instability^25,26^. Considerable research has examined the transient synchronous stability of REGCs under large disturbances^27^, primarily focusing on analysis, assessment, and improvement of the transient synchronous stability of single REGC units. Reference^28^ applied the phase-plane method to analyze the transient synchronous stability of REGCs, demonstrating that increasing the PLL regulation time and damping ratio can enhance REGC transient synchronous stability. In^29^, a Lyapunov function was constructed as a stability criterion for REGCs, providing reliable evaluation results. Reference^30^ proposed a method to improve REGC transient synchronous stability by compensating the active current using PLL frequency deviations, feeding back frequency variations during dynamic processes to adjust the post-fault active current reference.

Because both SVGs and REGCs employ GFL control, the transient synchronous stability of multi-converter systems consisting of SVGs and REGCs has also been investigated. Reference^31^ showed that increasing reactive current injection during severe disturbances can improve the transient synchronous stability of multi-converter systems. However, when VSCOM is integrated into renewable energy plants, the transient characteristics of VSCOM based on GFM control inevitably affect the synchronous stability of REGCs operating under GFL control during large disturbances^32^. To date, limited studies have explored the transient synchronous stability of mixed GFL-GFM multi-converter systems.

Reference^33^ conducted a preliminary investigation into the effect of GFL and GFM converters on the transient synchronous stability of purely inductive networks under severe disturbances, revealing that the stability of GFL devices depends on the q-axis voltage component, while the stability of GFM devices depends on the output electromagnetic power, with strong coupling between the two. Reference^34^ used a Lyapunov function and the equal-area criterion to analyze the effect of GFL-controlled REGC output currents on the transient synchronous stability of GFM-controlled converters. However, these studies did not specifically investigate the impact of GFM devices on the stability of GFL devices. In particular, the effect of a novel grid-forming reactive power compensator (VSCOM) on GFL-controlled REGCs, and the underlying mechanisms through which VSCOM enhances transient synchronous stability during large grid disturbances, remain unclear^4,35,36^.

To address the above-mentioned issues, a voltage-limit adaptive fault current control strategy for VSCOM is proposed, and the influence of VSCOM on the transient stability of renewable energy plants under weak grids is studied in this paper—thereby a theoretical and technical foundation is offered for the stable integration of large-scale renewable energy into weak grids. The main contributions are outlined as follows:

1. A novel voltage-source-based current limiting strategy is proposed to accommodate the operating characteristics of the VSCOM. This control scheme enables the VSCOM to operate at the maximum reactive current during grid faults and automatically adjusts the AC voltage reference, ensuring that the VSCOM maintains its GFM voltage source characteristics throughout the entire process.
2. A mathematical model of a renewable energy plant integrated with VSCOM is established. Analysis of this model demonstrates that the VSCOM significantly extends the static stability limit of the REGC in weak grids by clamping the voltage at the point of common coupling.
3. A transient synchronization stability mechanism model for the VSCOM-REGC system is developed. Utilizing the phase plane method, this study thoroughly uncover the coupling mechanism between the two, revealing that the VSCOM’s dynamics can dominate and stabilize the synchronization process of REGC.

The rest of the paper is organized as follows. In Section II, the control strategy based on voltage-limit adaptive fault current limit of VSCOM is proposed. The effect of VSCOM on the static stability limit of REGC is analyzed in Section III. The effect of VSCOM on the transient synchronous stability of REGC is studied is further presented in Section IV. Simulation analysis and conclusion are given in Sections V and VI.

## Connection method and control strategy of the virtual synchronous compensator

### Connection method of the virtual synchronous compensator

**Fig. 1:**
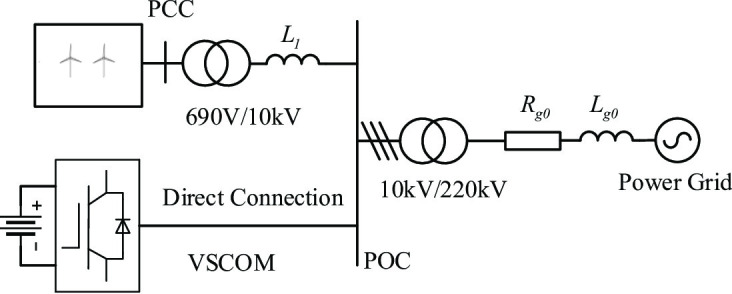
Connection method of VSCOM in a renewable energy power plant.

The connection configuration of the VSCOM in a renewable energy plant is illustrated in Fig. 1. The REGC is connected to the POC of the 10 kV collector substation through transformers and internal transmission lines. The VSCOM, functioning as a centralized reactive power compensator, is directly connected to the POC to provide voltage support for the renewable energy plant. The plant’s output is then delivered to the main grid via a step-up transformer at the collector substation and external transmission lines.

### Control strategy of the virtual synchronous compensator

**Fig. 2:**
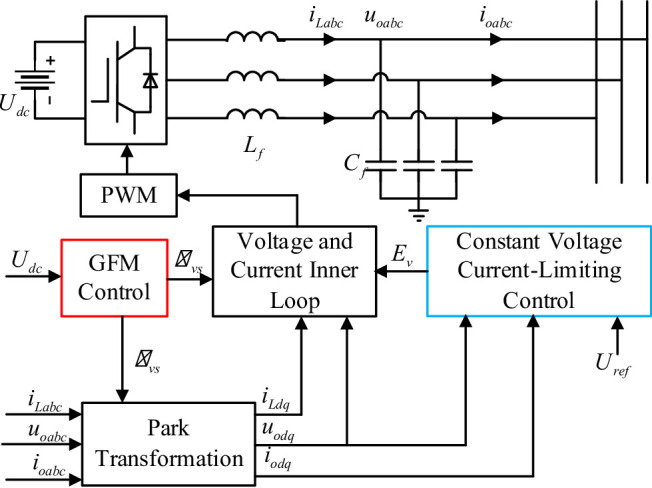
Control structure of the virtual synchronous compensator.

The control structure of the VSCOM is shown in Fig. 2. In the diagram, $\:{L}_{f}$ and $\:{C}_{f}$ represent the filter inductance and capacitance, respectively; $\:{U}_{\mathrm{dc}}$ is the DC-side voltage of the VSCOM; $\:{i}_{\mathrm{Labc}}$ is the inductor current; $\:{i}_{\mathrm{oabc}}$ and $\:{u}_{\mathrm{oabc}}$ are the output current and voltage of the VSCOM, respectively. The dq-axis components $\:{i}_{Ldq}$, $\:{i}_{odq}$, and $\:{u}_{odq}$ are obtained by applying the Park transformation to $\:{i}_{\mathrm{Labc}}$, $\:{i}_{\mathrm{oabc}}$, and $\:{u}_{\mathrm{oabc}}$, respectively. $\:{U}_{\mathrm{ref}}$ is the AC-side voltage reference, and $\:{\omega\:}_{vs}$ and $\:{\delta\:}_{vs}$ denote the output angular frequency and phase of the VSCOM.

The VSCOM control structure comprises two levels: outer-loop and inner-loop control. The outer-loop includes grid-forming synchronization control and constant voltage current-limiting control. The synchronization control generates the reference phase $\:{\theta\:}_{vs}$, ensuring synchronization between the VSCOM and the grid. The constant voltage current-limiting control provides the AC-side voltage reference $\:{E}_{v}$ for voltage support. The inner-loop implements closed-loop voltage and current control to precisely track the reference values generated by the outer-loop.

The VSCOM achieves GFM synchronization using virtual synchronous generator (VSG) technology^18^. In contrast to conventional PLL mechanisms, synchronization is accomplished without relying on PLL. The VSG control principle is described by the following equation:

1 $$\:J\frac{d{\omega\:}_{vs}}{dt}={P}_{vs}-{P}_{\mathrm{0}}-D({\omega\:}_{vs}-{\omega\:}_{0})$$

where $\:{\omega\:}_{0}$ is the nominal angular frequency, $\:{P}_{vs}$ is the active power output of the VSCOM, $\:{P}_{\mathrm{0}}$ is the reference active power, $\:J$ is the virtual inertia, and *D* is the damping coefficient of the VSCOM.

The active power of the VSCOM during grid transients is provided by the energy stored in its DC-side capacitor. Neglecting the capacitor’s own power loss, the transient active power delivered by the DC capacitor is equivalent to the VSCOM output active power and can be expressed as:

2 $$\:{P}_{vs}={P}_{\mathrm{dc}}=-{C}_{0}{U}_{\mathrm{dc}}\frac{d({U}_{\mathrm{dc}}-{U}_{\mathrm{dcref}})}{dt}$$

where $\:{U}_{\mathrm{dcref}}$ is the reference voltage of the DC-side capacitor, $\:{C}_{0}$ *i*s the DC-side capacitance, and $\:{P}_{\mathrm{dc}}$ is the active power output from the capacitor.

Since the VSCOM operates as a reactive power compensator, the steady-state active power reference is set to $\:{P}_{0}=0$. Combining (1) and (2), the synchronization control of the VSCOM based on DC-side voltage dynamics can be obtained as:

3 $$\:\left\{\begin{array}{c}J\frac{d{\omega\:}_{vs}}{dt}=-\frac{{C}_{0}}{2}\frac{d({U}_{\mathrm{dcref}}^{2}-{U}_{\mathrm{dc}}^{2})}{dt}-D({\omega\:}_{vs}-{\omega\:}_{0})\\\:{\theta\:}_{vs}=\int\:{\omega\:}_{vs}dt\end{array}\right.$$

Therefore, the VSCOM achieves grid synchronization through the dynamic response of the DC-side voltage. In addition to supporting plant voltage, this approach introduces additional system damping and enhances voltage stability beyond the capabilities of conventional SVG devices.

### Constant voltage Current-Limiting control of the virtual synchronous compensator

**Fig. 3:**
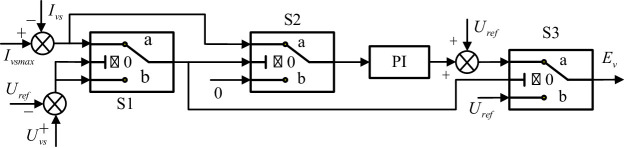
Low voltage ride-through (LVRT) control of the VSCOM.

With VSG control, the VSCOM can establish grid voltage directly at the POC, providing active voltage support. During normal operation, the VSCOM voltage reference $\:{E}_{v}$ is set to the nominal value $\:{U}_{\mathrm{ref}}$ and the reactive power output of the VSCOM adjusts automatically according to grid requirements. However, during grid faults that cause a significant voltage sag at the POC, maintaining a constant voltage output from the VSCOM can result in overcurrent. If current limiting is performed only in the inner-loop, the VSCOM may lose its grid-forming characteristics during faults^37^. Virtual impedance control can maintain the converter’s voltage source behavior but alters the impedance characteristics, which may affect the stability of grid-following renewable energy units when the VSCOM provides grid support^38^. To address this issue, this paper proposes a constant voltage current-limiting control strategy suitable for VSCOM. By dynamically adjusting the AC voltage reference, current limiting is achieved to prevent overcurrent during faults. Unlike traditional methods that alter impedance or directly truncate the current, this approach maintains the grid-forming voltage source characteristics of the VSCOM throughout the entire fault process, making it applicable to VSCOMs intended for voltage support. The control scheme is depicted in Fig. 3, where $\:{I}_{vs\mathrm{ref}}$ and $\:{I}_{vs}$ are the maximum allowable reactive current reference and the actual output current magnitude, respectively.

The specific control steps are as follows:

Step 1. During normal grid operation, the VSCOM operates in constant voltage mode, with $\:{U}_{vs}={U}_{\mathrm{ref}}$ and$\:\:{I}_{vs}<{I}_{vsmax}$. In this state, switches S1 (position a), S2 (position b), and S3 (position b) are engaged; the PI controller is inactive, and $\:{E}_{v}={U}_{\mathrm{ref}}$, maintaining a constant output voltage.

Step 2. At the moment of a grid voltage sag, the voltage source characteristic of the VSCOM temporarily keeps its output voltage constant, causing the output current to rise sharply and potentially exceed the maximum limit, thus activating the low voltage ride-through (LVRT) condition. Here, $\:{U}_{vs}={U}_{\mathrm{ref}}$ and $\:c$; switches S1 (position a), S2 (position a), and S3 (position a) are engaged, activating the PI controller to adjust the voltage reference and regulate the output current to within the allowable limit.

Step 3. During the voltage sag period, the VSCOM operates with a reduced voltage reference but maintains maximum current output, that is, $\:{U}_{vs}<{U}_{\mathrm{ref}}$ and $\:{I}_{vs}={I}_{vsmax}$. Switches S1 (position b), S2 (position a), and S3 (position a) are engaged, and the VSCOM voltage reference is still generated by the PI controller.

Step 4. After the fault clears and grid voltage recovers, $\:{U}_{vs}={U}_{\mathrm{ref}}$ and $\:{I}_{vs}<{I}_{vsmax}$, and the VSCOM returns to the initial constant voltage mode.

## Analysis of the effect of VSCOM on the static stability limit of REGC

**Fig. 4:**
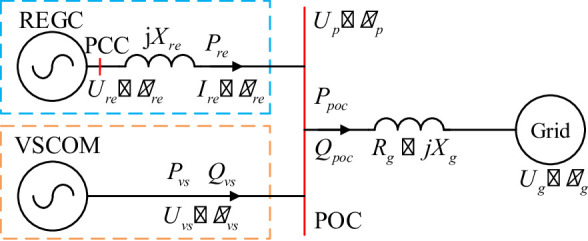
Equivalent circuit of the VSCOM grid connection.

This section analyzes the voltage support capability of the VSCOM under weak grid conditions and its effect on the static stability limit of the REGC, based on the control strategy described previously. The equivalent mechanism model of a renewable energy plant with VSCOM, corresponding to Fig. 1, is shown in Fig. 4.

In this model, the REGC is represented as a current source with output current magnitude $\:{I}_{\mathrm{re}}$ and phase angle $\:{\theta\:}_{re}$, while the VSCOM is modeled as a voltage source with output voltage magnitude $\:{U}_{vs}$ and phase angle $\:{\theta\:}_{vs}$. The POC voltage has a magnitude $\:{U}_{p}$ and phase $\:{\theta\:}_{p}$. The grid and transmission lines are represented as a voltage source with magnitude $\:{U}_{g}$ and phase $\:{\theta\:}_{g}$, in series with impedance $\:{R}_{g}+j{X}_{g}$. The total impedance $\:{X}_{re}$ is the sum of the transmission line and transformer impedance within the renewable energy plant. $\:{P}_{re}$ and $\:{Q}_{vs}$ denote the active power output of the renewable energy source and the reactive power output of the VSCOM, respectively. $\:{P}_{poc}$ and $\:{Q}_{poc}$ are the active and reactive power outputs of the entire renewable energy plant at the POC. For the following analysis, the REGC is assumed to supply only active power, with all reactive power support provided by the VSCOM. Converter losses are neglected.

In the absence of the VSCOM, the voltage magnitude at the REGC terminal $\:{U}_{re}$ is given by:

4 $$\:{U}_{\mathrm{re}}={I}_{\mathrm{re}}{R}_{g}+\sqrt{{U}_{g}^{2}-({X}_{g}+{X}_{\mathrm{re}}{)}^{2}{I}_{\mathrm{re}}^{2}}$$

When the REGC outputs only active power, its actual active power $\:{P}_{re}$ can be expressed as:

5 $$\:{P}_{\mathrm{re}}=1.5{U}_{\mathrm{re}}{I}_{\mathrm{re}}$$

From (4) and (5), the relationship between the REGC active power output Pre and the AC-side terminal voltage $\:{U}_{re}$ without VSCOM can be derived as:

6 $$\:1.5{U}_{\mathrm{re}}^{2}={P}_{\mathrm{re}}{R}_{g}+\sqrt{2.25{U}_{\mathrm{re}}^{2}{U}_{g}^{2}-({X}_{g}+{X}_{\mathrm{re}}{)}^{2}{P}_{\mathrm{re}}^{2}}$$

After VSCOM integration, as shown in Fig. 4, if the VSCOM operates in an unsaturated state, the POC voltage remains constant, and the REGC terminal voltage $\:{U}_{re}$ can be expressed as:

7 $$\:{U}_{\mathrm{re}}=\sqrt{{U}_{p}^{2}-{X}_{\mathrm{re}}^{2}{I}_{\mathrm{re}}^{2}}=\sqrt{{U}_{\mathrm{ref}}^{2}-{X}_{\mathrm{re}}^{2}{I}_{\mathrm{re}}^{2}}$$

In typical configurations, the line impedance $\:{X}_{\mathrm{re}}$ between the REGC and the POC is small, so $\:{U}_{\mathrm{re}}\approx\:{U}_{p}={U}_{\mathrm{ref}}$. Therefore, under unsaturated conditions, the REGC terminal voltage remains stable.

Once the VSCOM reaches saturation, it operates in constant voltage current-limiting mode, delivering its maximum reactive current for compensation. Under this condition, the system configuration in Fig. 4 is analyzed using the voltage phase angle at the POC as the reference, where *θ* denotes the voltage phase difference between the PCC and the POC. The following equations are established:

8 $$\:{U}_{p}=({I}_{re}{\angle\:}\theta\:-j{I}_{\mathrm{vs}max})(j{X}_{g}+{R}_{g})+{U}_{g}{\angle\:}{\theta\:}_{g}$$

9 $$\:{U}_{re}\angle\:\theta\:={I}_{d}\angle\:\theta\:\cdot\:j{X}_{re}+{U}_{p}$$

Expanding (8) and (9) and separating the real and imaginary components yields:

10 $$\:{U}_{p}={I}_{re}{R}_{g}\mathrm{c}\mathrm{o}\mathrm{s}\theta\:-{I}_{re}{X}_{g}\mathrm{s}\mathrm{i}\mathrm{n}\theta\:+{{I}_{\mathrm{vs}max}X}_{g}+{U}_{g}\mathrm{c}\mathrm{o}\mathrm{s}{\theta\:}_{g}$$

11 $$\:0={I}_{re}{X}_{g}cos\theta\:+{I}_{re}{R}_{g}sin\theta\:-{I}_{\mathrm{vs}max}{R}_{g}+{U}_{g}sin{\theta\:}_{g}$$

12 $$\:{U}_{re}cos\theta\:={U}_{p}-{I}_{re}{X}_{re}sin\theta\:\:$$

13 $$\:{U}_{re}sin\theta\:={I}_{re}{X}_{re}cos\theta\:$$

From (4), the grid voltage phase angle is determined as:

14 $$\:{\theta\:}_{g}=-\mathrm{a}\mathrm{r}\mathrm{c}\mathrm{t}\mathrm{a}\mathrm{n}\left(\frac{{I}_{re}{X}_{g}cos\theta\:+{I}_{re}{R}_{g}sin\theta\:-{I}_{\mathrm{vs}max}{R}_{g}}{{U}_{g}}\right)$$

Equation (6) provides the expression for the phase difference:

15 $$\:\theta\:=arctan\frac{{I}_{re}{X}_{re}}{{U}_{re}}$$

Substituting (10) into (12) gives:

16 $$\:{U}_{re}cos\theta\:={I}_{re}{R}_{g}cos\theta\:-{I}_{re}{(X}_{g}+{X}_{re})sin\theta\:+{{I}_{\mathrm{vs}max}X}_{g}+{U}_{g}cos{\theta\:}_{g}$$

Further substitution of (14) and (15) into Eq. (16) leads to:

$$\:{U}_{\mathrm{re}}\frac{{U}_{re}}{\sqrt{{U}_{re}^{2}+{I}_{re}^{2}{X}_{re}^{2}}}={I}_{\mathrm{re}}{R}_{g}\frac{{U}_{re}}{\sqrt{{U}_{re}^{2}+{I}_{re}^{2}{X}_{re}^{2}}}+{I}_{\mathrm{vs}max}{X}_{g}-\frac{{I}_{\mathrm{re}}^{2}\left({X}_{g}+{X}_{\mathrm{re}}\right){X}_{\mathrm{re}}}{{U}_{\mathrm{re}}}\frac{{I}_{re}{X}_{re}}{\sqrt{{U}_{re}^{2}+{I}_{re}^{2}{X}_{re}^{2}}}$$

17 $$\:+{U}_{g}\sqrt{1-(\frac{\frac{{I}_{re}{X}_{g}{U}_{re}}{\sqrt{{U}_{re}^{2}+{I}_{re}^{2}{X}_{re}^{2}}}+\frac{{I}_{re}{R}_{g}{I}_{re}{X}_{re}}{\sqrt{{U}_{re}^{2}+{I}_{re}^{2}{X}_{re}^{2}}}-{I}_{\mathrm{vs}max}{R}_{g}}{{U}_{g}}{)}^{2}\:\:\:\:\:\:}$$

After algebraic rearrangement, the final expression is obtained as:

18 $$\:{U}_{\mathrm{re}}={I}_{\mathrm{re}}{R}_{g}+{I}_{\mathrm{vs}max}{X}_{g}-\frac{{I}_{\mathrm{re}}^{2}\left({X}_{g}+{X}_{\mathrm{re}}\right){X}_{\mathrm{re}}}{{U}_{\mathrm{re}}}+\sqrt{{U}_{g}^{2}-({X}_{g}{I}_{\mathrm{re}}-{I}_{\mathrm{vs}max}{R}_{g}+{\frac{{I}_{\mathrm{re}}^{2}{R}_{g}{X}_{\mathrm{re}}}{{U}_{\mathrm{re}}})}^{2}}$$

Combining (5) and (18), the relationship between $\:{P}_{\mathrm{re}}$ and $\:{U}_{\mathrm{re}}$ after VSCOM integration is:

19 $$\:{U}_{\mathrm{re}}=\frac{{P}_{\mathrm{re}}{R}_{g}}{1.5{U}_{\mathrm{re}}}+{I}_{\mathrm{vs}max}{X}_{g}-\frac{{P}_{\mathrm{re}}^{2}({X}_{g}+{X}_{\mathrm{re}}){X}_{\mathrm{re}}}{2.25{U}_{\mathrm{re}}^{3}}+\sqrt{{U}_{g}^{2}-(\frac{{P}_{\mathrm{re}}{X}_{g}}{1.5{U}_{\mathrm{re}}}-{I}_{\mathrm{vs}max}{R}_{g}+{\frac{{P}_{\mathrm{re}}^{2}{R}_{g}{X}_{\mathrm{re}}}{2.25{U}_{\mathrm{re}}^{3}})}^{2}}$$

**Fig. 5:**
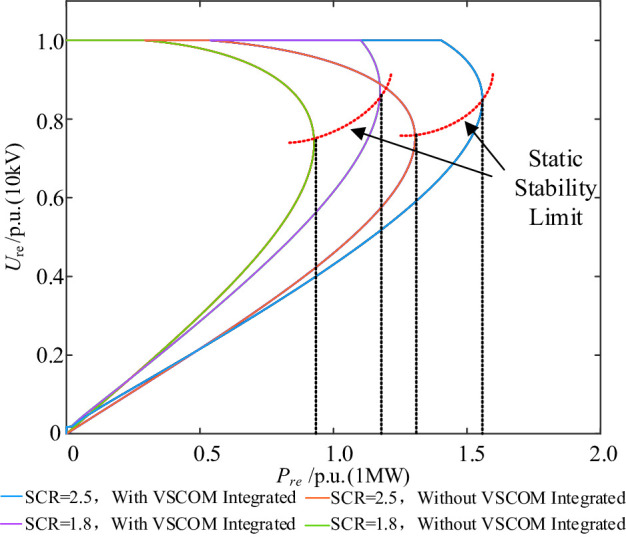
*P*_*re*_*-U*_*re*_ curve before and after VSCOM integration under different SCR conditions.

The *P*_*re*_*-U*_*re*_ curves in Fig. 5 indicate that, under weak grid conditions, the REGC has a limited maximum active power output, which is highly dependent on the SCR. A lower SCR reduces the maximum achievable output power of the REGC. For example, with SCR = 2.5, the maximum REGC output is 1.305 p.u., whereas under extremely weak grid conditions (SCR = 1.8), the REGC output cannot reach 1.0 p.u. and is limited to 0.927 p.u. This is due to the increased equivalent grid reactance associated with reduced SCR, which enhances the coupling between the active current component and grid reactance. As the REGC active power increases, the terminal voltage of the renewable energy source declines, further limiting REGC power output.

Additionally, Fig. 5 shows that VSCOM integration at the POC stabilizes both the POC voltage and the REGC terminal voltage. After VSCOM integration, the *P*_*re*_*-U*_*re*_ curve can be divided into two segments: the first is a linear segment, where the VSCOM, acting as a voltage source, maintains the POC voltage at 1 p.u. within its reactive compensation capacity, enabling the REGC to operate at rated active power output; the second is a nonlinear segment, where further increases in REGC active power output $\:{P}_{\mathrm{re}}$ cause the REGC terminal voltage $\:{U}_{\mathrm{re}}$ to decrease gradually.

Moreover, VSCOM integration significantly enhances the maximum output power capability of the REGC. When SCR = 2.5, the maximum REGC output rises to 1.557 p.u.; even under extremely weak grid conditions (SCR = 1.8), the REGC output exceeds 1.0 p.u., reaching 1.174 p.u., as shown in Fig. 5. These results demonstrate that under weak and extremely weak grid conditions, VSCOM, operating as a voltage source, effectively increases the static stability limit of the REGC and ensures voltage stability at the REGC terminal during rated output.

## Analysis of the effect of VSCOM on the transient synchronous stability of REGC under grid faults

The previous section showed that integration of the VSCOM significantly enhances the static stability limit of the REGC. To further examine how VSCOM affects the transient stability of REGC, this section develops a transient model for a renewable energy plant with VSCOM and applies the phase-plane method to analyze the effect of VSCOM integration at the POC on the transient synchronous stability of the REGC. Since the control bandwidth of the voltage and current inner loops in both the VSCOM and REGC is far greater than that of their outer synchronization control loops, the influence of the inner-loop dynamics is neglected in the subsequent analysis of the transient synchronization stability between the VSCOM and REGC^27–34^.

### Analysis of the Mechanism of VSCOM Access to the Transient Impact on REGC

Based on the equivalent circuit in Fig. 4, the REGC terminal voltage can be expressed as:

20 $$\:{U}_{\mathrm{re}}{e}^{j{\theta\:}_{\mathrm{re}}}={X}_{\mathrm{re}}{I}_{\mathrm{re}}{e}^{j({\theta\:}_{\mathrm{re}}+\pi\:\mathrm{/2})}+{U}_{vs}{e}^{j{\theta\:}_{vs}}$$

A conventional REGC uses a PLL to extract the terminal voltage phase $\:{\theta\:}_{\mathrm{re}}$ for grid synchronization. The PLL dynamics are described by:

21 $$\:{\theta\:}_{\mathrm{re}}=\int\:({\omega\:}_{0}+{K}_{p}{U}_{\mathrm{re},q}+{K}_{i}\int\:{U}_{\mathrm{re},q}dt)dt$$

where $\:{K}_{p}$ and $\:{K}_{i}$ are the proportional and integral gains of the PLL, respectively, and $\:{U}_{\mathrm{re},q}$ is the q-axis component of the REGC terminal voltage obtained via Park transformation.

Using the PLL output angle $\:{\theta\:}_{\mathrm{re}}$ as the Park transformation reference, $\:{U}_{\mathrm{re},q}$ can be expressed as:

22 $$\:{U}_{\mathrm{re,}q}={X}_{\mathrm{re}}{I}_{\mathrm{re}}+{U}_{\mathrm{vs}}\mathrm{sin}({\theta\:}_{vs}-{\theta\:}_{\mathrm{re}})$$

By rearranging (2), the relationship between the VSCOM active power output and the DC voltage during transients is:

23 $$\:{U}_{\mathrm{dc}}^{2}=\int\:-\frac{2}{{C}_{0}}{P}_{vs}dt+{U}_{\mathrm{dcref}}^{2}$$

According to Fig. 4, the transient active power output of the VSCOM is:

24 $$\:{P}_{vs}={P}_{\mathrm{poc}}-{P}_{\mathrm{re}}={P}_{\mathrm{poc}}-1.5{U}_{vs}{I}_{\mathrm{re}}$$

The active power $\:{P}_{\mathrm{poc}}$ transmitted from the POC to the grid is:

25 $$\:{P}_{\mathrm{poc}}=\frac{3}{2}\frac{{R}_{g}{{U}_{vs}}^{2}-{R}_{g}{U}_{g}{U}_{vs}{cos}{\theta\:}_{vs}+{X}_{g}{U}_{g}{U}_{\mathrm{vs}}{sin}{\theta\:}_{vs}}{{R}_{g}^{2}+{X}_{g}^{2}}$$

The POC voltage magnitude is equal to the VSCOM output voltage:

26 $$\:\left\{\begin{array}{c}{U}_{p}={U}_{vs}={E}_{0},{I}_{\mathrm{vs}}<{I}_{\mathrm{vs}max}\\\:{U}_{p}={U}_{vs}={I}_{\mathrm{re}}{R}_{g}+{I}_{\mathrm{vs}max}{X}_{g}\\\:+\sqrt{{U}_{g}^{2}-{({X}_{g}{I}_{\mathrm{re}}-{I}_{\mathrm{vs}max}{R}_{g})}^{2}},{I}_{\mathrm{vs}}={I}_{\mathrm{vs}max}\end{array}\right.\:\:\:\:\:\:\:\:\:\:\:\:\:\:\:\:\:\:\:\:\:\:\:\:\:\:\:\:\:\:\:\:\:\:\:\:\:\:\:\:$$

**Fig. 6:**
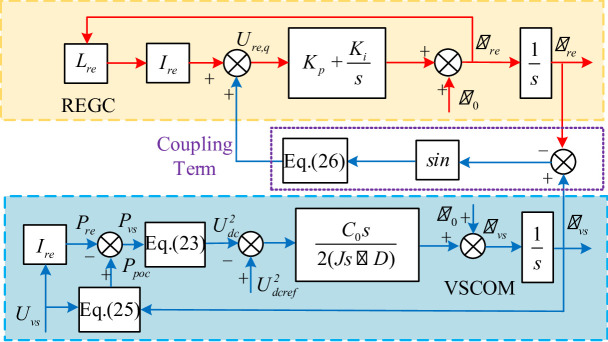
Transient model of joint operation of VSCOM with renewable energy plant.

Combining (3), (21), (22), (23), (24), (25), and (26), the joint transient model of the renewable energy plant with integrated VSCOM and REGC is obtained, as shown in Fig. 6.

Inspection of (22) reveals two terms; where the second term represents the coupling term effect between VSCOM and REGC, as illustrated in Fig. 6. The transient model indicates that VSCOM introduces a new dynamic path (highlighted in blue) that directly affects the transient stability of the REGC.

Analysis indicates that System disturbances first affect the VSCOM, and its transient characteristics are then transferred to the REGC. Furthermore, because $\:{X}_{\mathrm{re}}{I}_{\mathrm{re}}\ll\:{U}_{\mathrm{vs}}$, and the VSCOM synchronization loop responds is slower than that of REGC’s PLL, causing the transient behavior of REGC to be dominated by VSCOM. This means that after VSCOM integration, the REGC—originally operating in grid-following mode—exhibits partial grid-forming characteristics.

When grid voltage sags, the proportional term of the PLL controller causes an abrupt change in the REGC output angular frequency $\:{\omega\:}_{\mathrm{re}}$, while the output phase $\:{\theta\:}_{\mathrm{re}}$ does not change immediately due to the integrator. From (22), the steady-state phase difference between the VSCOM and the renewable energy source is:

27 $$\:{\theta\:}_{vs}-{\theta\:}_{\mathrm{re}}=-{arcsin}\frac{{X}_{\mathrm{re}}{I}_{\mathrm{re}}}{{U}_{vs}}$$

Substituting (27) and (22) into (21) yields the frequency jump $\:\varDelta\:{\omega\:}_{re,vs}$ of the REGC with VSCOM integration:

28 $$\:{\Delta\:}{\omega\:}_{re,vs}=\frac{\Delta{U}_{vs}{K}_{p}{\omega\:}_{g}{L}_{\mathrm{re}}{I}_{\mathrm{re}}}{{U}_{\mathrm{ref}}(1-{K}_{p}{L}_{\mathrm{re}}{I}_{\mathrm{re}})}$$

where $\:{L}_{\mathrm{re}}={X}_{\mathrm{re}}/{\omega\:}_{\mathrm{re}}$ is the equivalent line reactance between the REGC and the POC, and $\:{\Delta\:}{U}_{re}$ is the voltage magnitude difference of the VSCOM output before and after the fault, calculated by (26).

Without VSCOM integration, the sudden change $\:{\Delta\:}{\omega\:}_{\mathrm{re}}$ of the output angular frequency of REGC caused by the grid voltage sag can be expressed as:

29 $$\Delta{\omega\:}_{\mathrm{re}}=\frac{\Delta{U}_{g}{K}_{p}{sin}{\theta\:}_{\mathrm{re}}}{1-{K}_{p}({L}_{g}+{L}_{\mathrm{re}}){I}_{\mathrm{re}}}$$

where $\triangle{U}_{g}$ is the grid voltage magnitude difference before and after the fault.

After the fault, $\triangle{U}_{g}\approx\triangle{U}_{\mathrm{vs}}$, the ratio of frequency jumps is:

30 $$\:\frac{\Delta{\omega\:}_{re,vs}}{\Delta{\omega\:}_{\mathrm{re}}}=\frac{{L}_{\mathrm{re}}{I}_{\mathrm{re}}{\omega\:}_{g}[1-{K}_{p}({L}_{g}+{L}_{\mathrm{re}}\left){I}_{\mathrm{re}}\right]}{{sin}{\theta\:}_{\mathrm{re}}{U}_{\mathrm{ref}}(1-{K}_{p}{L}_{\mathrm{re}}{I}_{\mathrm{re}})}$$

**Fig. 7:**
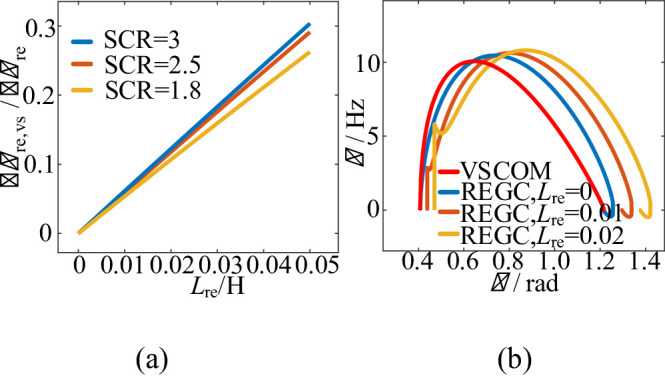
Influence of$\:{L}_{\mathrm{re}}$ on REGC transient response under large grid disturbances. (**a**) Effect of $\:{L}_{\mathrm{re}}$ on $\triangle{\omega\:}_{\mathrm{re,vs}}/\triangle{\omega\:}_{\mathrm{re}}$. (**b**) REGC phase-plane under different $\:{L}_{\mathrm{re}}$ values.

According to (20), the effect of VSCOM on the frequency deviation of REGC during transients can be quantified. Figure 7(a) shows that $\triangle{\omega\:}_{re,vs}/\triangle{\omega\:}_{\mathrm{re}}$ increases with $\:{L}_{\mathrm{re}}$ but remains less than 1. Notably, when $\:{L}_{\mathrm{re}}=0$, implying negligible internal line impedance, then $\triangle{\omega\:}_{re,vs}=0$. This indicates that the VSCOM clamps the REGC frequency, preventing abrupt frequency deviations during grid faults. Therefore, VSCOM integration reduces REGC frequency jump of REGC during grid faults and enhances its transient stability.

From Fig. 6, the nonlinear dynamic equation for the REGC with VSCOM integration can be derived, as shown in (31):

31 $$\:\left\{\begin{array}{c}{\omega\:}_{\mathrm{re}}={\omega\:}_{0}+\Delta{\omega\:}_{\mathrm{re}}={\omega\:}_{0}+{\dot{\theta\:}}_{\mathrm{re}}\\\:{\omega\:}_{vs}={\omega\:}_{0}+\Delta{\omega\:}_{vs}={\omega\:}_{0}+{\dot{\theta\:}}_{\mathrm{vs}}\\\:{\ddot{\theta\:}}_{\mathrm{re}}={K}_{i}{U}_{vs}\mathrm{s}\mathrm{i}\mathrm{n}({\theta\:}_{vs}-{\theta\:}_{\mathrm{re}})+{K}_{i}({\omega\:}_{0}+{\dot{\theta\:}}_{\mathrm{re}}){L}_{\mathrm{re}}{I}_{\mathrm{re}}\\\:+{K}_{p}{U}_{vs}({\dot{\theta\:}}_{vs}-{\dot{\theta\:}}_{\mathrm{re}})\mathrm{c}\mathrm{o}\mathrm{s}({\theta\:}_{vs}-{\theta\:}_{\mathrm{re}})+{K}_{p}{\dot{\theta\:}}_{\mathrm{re}}{L}_{\mathrm{re}}{I}_{\mathrm{re}}\\\:J{\ddot{\theta\:}}_{vs}={P}_{\mathrm{poc}}-{P}_{\mathrm{re}}-D{\dot{\theta\:}}_{vs}\end{array}\right.$$

Equation (31) shows that the transient behavior of the REGC is coupled with that of the VSCOM, and depends on the REGC output current, PLL parameters, VSCOM voltage magnitude and phase, and the electrical distance $\:{L}_{\mathrm{re}}$ between REGC and VSCOM. Compared with PLL-based synchronization, VSCOM provides higher damping and equivalent inertia due to grid-forming control, which enhances stability during transients.

When$\:\:{L}_{\mathrm{re}}=0$, the transient behavior of the REGC is mainly determined by PLL parameters and VSCOM voltage characteristics. As shown in Fig. 7(b), under these conditions, strong coupling occurs between the REGC and VSCOM, and the REGC phase trajectory closely follows that of the VSCOM.

In practical systems where $\:{L}_{\mathrm{re}}\ne\:0$, the REGC exhibits frequency jumps at the onset of faults, and the phase difference between the REGC and VSCOM increases due to $\:{L}_{\mathrm{re}}$. As $\:{L}_{\mathrm{re}}$ increases, the influence of VSCOM on the REGC weakens, the REGC phase-plane trajectory shifts to the upper right, and transient stability is reduced.

Therefore, the degree of coupling between VSCOM and REGC is mainly determined by $\:{L}_{\mathrm{re}}$. When $\:{L}_{\mathrm{re}}$ is small, strong coupling occurs, allowing the VSCOM to dominate the transient behavior of the REGC. This is favorable for enhancing transient synchronous stability under weak grid conditions.

### Analysis of the influence of VSCOM parameters and grid characteristics on the transient stability of renewable energy systems

#### Influence of VSCOM on the transient stability of renewable energy under different grid Short-Circuit ratios

Assuming the grid voltage drops to 0.4 p.u., the phase-plane diagrams of the REGC before and after VSCOM integration under different SCRs are obtained using (25). The parameters used in the analysis are listed in Table 1.

**Fig. 8:**
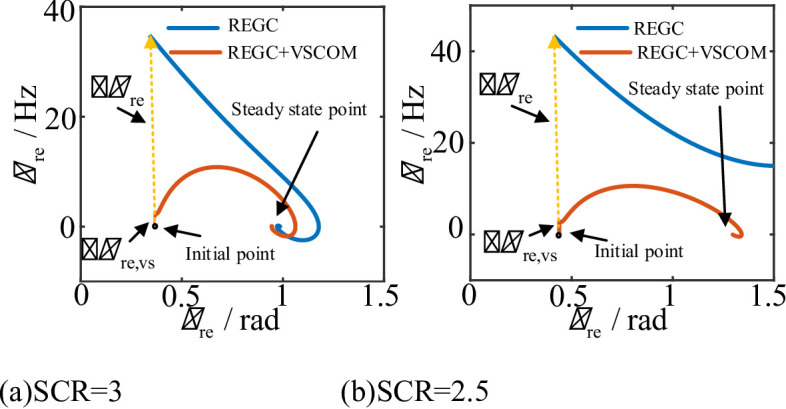
Phase-plane diagrams of renewable energy units before and after VSCOM integration under different SCRs. (**a**) SCR = 3, (**b**) SCR = 2.5.

As shown in Fig. 8(a), when the grid SCR is 3, the REGC remains stable and converges to a new equilibrium point regardless of whether the VSCOM is integrated. However, with VSCOM integration, the new steady-state point shifts downward relative to the case without VSCOM, reflecting the voltage support and reactive power compensation provided by VSCOM.

Additionally, Fig. 8(a) illustrates the degree of frequency step change in renewable energy output during grid transients, as described by (28) and (29). Without VSCOM, the REGC experiences a significant frequency jump at the onset of the voltage sag, followed by gradual damping and stabilization. After VSCOM integration, the REGC frequency exhibits only a minor step change during the transient, and the peak frequency is much lower than in the independent REGC operation case. This improvement is attributed to the grid-forming control of the VSCOM, which provides higher equivalent inertia and effectively suppresses frequency fluctuations during grid disturbances.

As shown in Fig. 8(b), when the grid SCR is 2.5, the REGC experiences transient instability. This instability arises because the intrinsic damping of the REGC is insufficient, preventing it from reaching a new equilibrium point. However, after the VSCOM is connected in parallel at the POC, the REGC maintains stability even under voltage sag conditions with a low SCR. This is due to the VSCOM, operating with grid-forming control, providing sufficient system damping and voltage support, thereby ensuring the transient stability of the REGC.

#### Influence of VSCOM damping and inertia coefficients on the transient stability of renewable energy

**Fig. 9:**
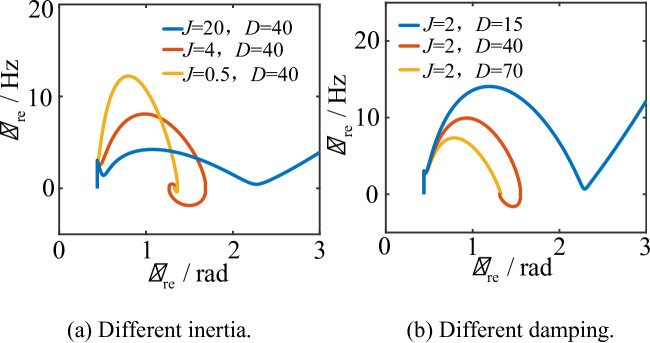
Phase-plane diagrams of renewable energy power units under different VSCOM damping *D* and inertia *J.* (**a**) Different inertia, (**b**) Different damping.

Figure 9 presents the phase-plane diagrams of the REGC with VSCOM integration under different damping and inertia coefficients when the grid voltage drops to 0.4 p.u.

As shown in Fig. 9, the VSCOM inertia *J* and damping *D* do not affect the initial frequency step change of the renewable energy source. Increasing the inertia *J* slows the rate of frequency variation, extending the system acceleration process and improving the dynamic response speed. However, excessive *J* leads to greater overshoot in the output phase angle of the renewable energy source. For example, when *J* = 20, the system remains in an acceleration phase throughout the transient process, causing continuous growth of the power angle and ultimately leading to transient instability of the renewable energy system. Therefore, selecting *J* requires balancing the frequency response speed and overall system stability.

The effect of the VSCOM damping coefficient *D* on transient stability is illustrated in Fig. 9(b). When *D* = 70, the transient response of the renewable energy source exhibits no overshoot. When *D* = 40, overshoot appears in the transient response of the power angle. When *D* = 15, insufficient damping cannot suppress continuous frequency acceleration, resulting in transient instability. Thus, increasing the damping coefficient *D* contributes to improving system stability.

For VSCOM-integrated renewable energy systems, the transient response of the renewable energy source is largely influenced by the inertia *J* and damping *D* of the VSCOM. In fact, during independent grid connection of renewable energy units, the equivalent damping of PLL-synchronized converters is negatively correlated with the output phase angle $\:{\theta\:}_{\mathrm{re}}$. As $\:{\theta\:}_{\mathrm{re}}$ increases, the equivalent damping decreases and may become negative, making independently operated renewable energy systems susceptible to transient stability issues due to insufficient damping. By adjusting the VSCOM damping coefficient *D*, the system can maintain significant positive damping, which substantially improves the transient stability of renewable energy sources. Therefore, increasing *D* and reducing *J* effectively enhance the transient stability of the renewable energy system.

#### Influence of VSCOM reactive power capacity on the transient stability of renewable energy

**Fig. 10:**
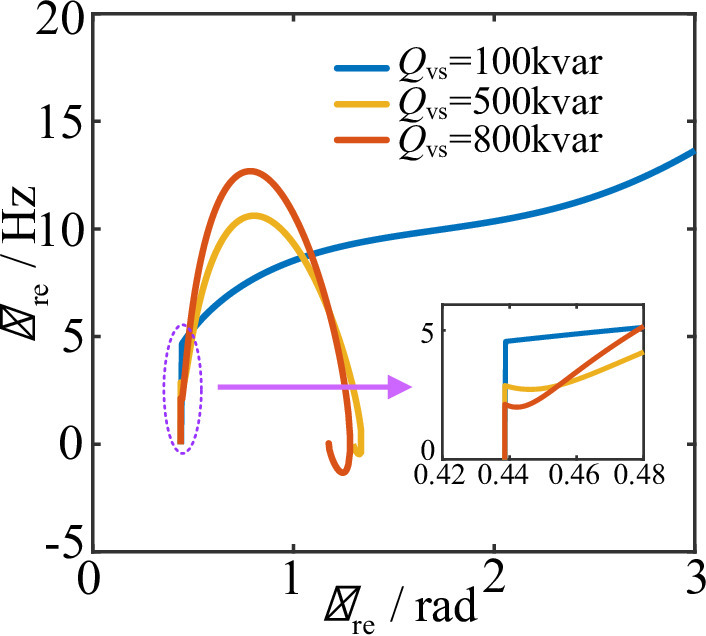
Phase-plane diagrams of renewable energy power units under different VSCOM reactive power capacities.

Figure 10 shows the phase-plane diagrams of the REGC under different VSCOM reactive power capacities when the grid voltage drops to 0.4 p.u. As illustrated, when the VSCOM reactive power capacity $\:{Q}_{vs}=100kvar$, the REGC experiences transient instability. As $\:{Q}_{vs}$ increases, the REGC can stably reach a new equilibrium point. According to (30), increasing the VSCOM reactive power capacity improves the terminal voltage of both the REGC and VSCOM during faults. From (28), higher REGC terminal voltage reduces the magnitude of the frequency step change during disturbances.

Additionally, based on (30), increasing the VSCOM terminal voltage $\:{U}_{vs}$ strengthens the coupling between the renewable energy source and the VSCOM, causing the PLL output angle $\:{\theta\:}_{\mathrm{re}}$ of the REGC to more closely follow the VSCOM phase angle $\:{\theta\:}_{vs}$. Therefore, the transient stability of the REGC becomes more dependent on the dynamic response of the VSCOM. Therefore, increasing the reactive power capacity of the VSCOM is an effective way to improve the transient stability of renewable energy systems.

## Simulation analysis

To verify the effectiveness of the proposed constant voltage current-limiting strategy for the VSCOM and the influence of VSCOM on the transient synchronous stability of the REGC, a detailed electromagnetic transient simulation model of the REGC and VSCOM was developed based on the system configuration in Fig. 1 using MATLAB/Simulink. The key simulation parameters for the REGC and VSCOM are provided in Table 1.

**Table 1:** REGC and VSCOM simulation parameters.

Parameter	Value	Parameter	Value
Grid line voltage (rms) *U*_*g*_/kV	10	Rated reactive power capacity of VSCOM *Q*_*vs.*_/Mvar	0.3
Grid inductance *L*_*g*_/mH) for SCR = 3/2.5/1.8	106/127/177	Low-voltage ride-through controller gains *K*_*lvrt, vp*_, *K*_*lvrt, vi*_	0.7,2592
Grid resistance *R*_*g*_/Ω	5	Virtual inertia *J* and damping coefficient *D*	1,50
Equivalent internal line inductance of renewable energy plant *L*_*re*_/mH	10	VSCOM filter inductance *L*_*fvs*_/mH	63.7
Grid angular frequency *ω*_*g*_/(rad/s)	314	VSCOM filter resistance *R*_*fvs*_/Ω	0.04
Active power setpoint of REGC *P*_*re*_/MW	1	VSCOM filter capacitance *C*_*fvs*_/µF	2.53
REGC-PLL controller gains *K*_*cp.*_, *K*_*ci*_	0.018,0.32	DC-side capacitance *C*_0_/mF	7
REGC filter inductance *L*_*fc*_/mH	111	DC-side voltage *U*_*dc*_/kV	15
REGC filter resistance *R*_*fc*_/Ω	0.056		

### Validation of VSCOM constant voltage Current-Limiting control

To verify the effectiveness of the proposed constant voltage current-limiting control for the VSCOM, a grid voltage sag to 0.6 p.u. was applied in the simulation model.

**Fig. 11:**
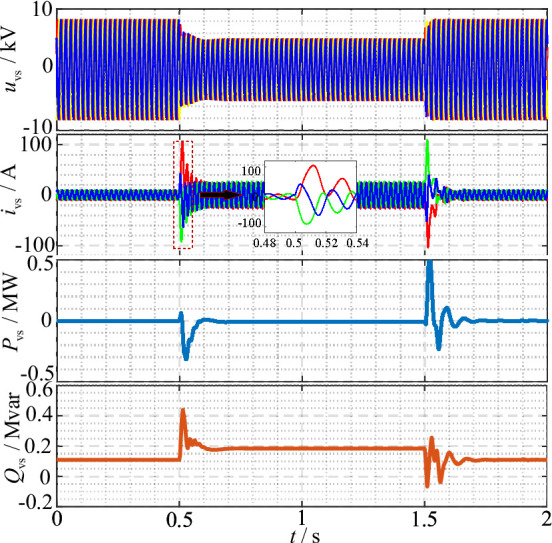
VSCOM output voltage, current, active power, and reactive power during a grid voltage sag to 0.6 p.u.

As shown in Fig. 11, before the grid fault, the VSCOM maintained constant voltage output with zero active power. At 0.5 s, when the grid voltage dropped, the current-limiting control of the VSCOM became active, proactively reducing the output voltage to an appropriate level while maintaining saturated reactive current output to support the grid. Due to the delay in adjusting the reference voltage to its optimal value immediately after the fault, a short overcurrent—lasting approximately two cycles—was observed. However, stable operation was quickly restored. During the transient, to maintain synchronization with the grid, the grid-forming synchronization control based on DC voltage regulation induced slight fluctuations in the VSCOM active power, supplied from the DC-side capacitor. This phenomenon is analogous to the VSCOM providing additional inertia to the grid. After a brief transient, the VSCOM resumed steady-state operation with zero active power output.

### Validation of the effect of VSCOM on the static stability limit of REGC

Based on the static stability analysis in Sect. "Connection method and control strategy of the virtual synchronous compensator", the enhancement effect of VSCOM on the static stability limit of the REGC under extremely weak grid conditions (SCR = 1.8) was verified. The active power setpoint of the REGC was first set near the critical stability limit. At 0.5 s, the setpoint was gradually increased by 20 kW/s to approach the static stability boundary, continuing until the setpoint exceeded the stability limit, resulting in instability.

**Fig. 12:**
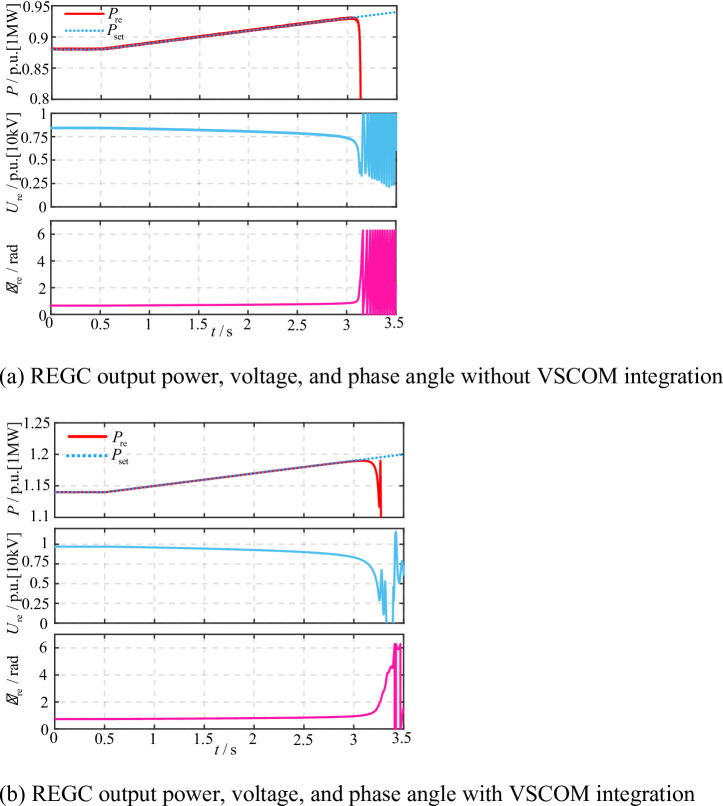
Impact of VSCOM on the static stability limit of renewable energy under SCR = 1.8. (**a**) REGC output power, voltage, and phase angle without VSCOM integration, (**b**) REGC output power, voltage, and phase angle with VSCOM integration.

As shown in Fig. 12(a), with SCR = 1.8, the REGC maintained stable output as active power increased, but instability occurred when the setpoint reached approximately 0.93 p.u., consistent with the theoretical analysis in Sect. 2. With VSCOM integration, Fig. 12(b) demonstrates that the REGC output limit increased to approximately 1.18 p.u., representing a 26.9% improvement. Furthermore, without VSCOM, the REGC terminal voltage near the critical output was only 0.85 p.u., while VSCOM integration raised the terminal voltage to approximately 1.0 p.u. These results confirm that VSCOM significantly improves both the static stability and the maximum stable output of the REGC under extremely weak grid conditions.

### Validation of the influence of VSCOM on the transient synchronous stability of REGC under weak grid conditions

**Fig. 13:**
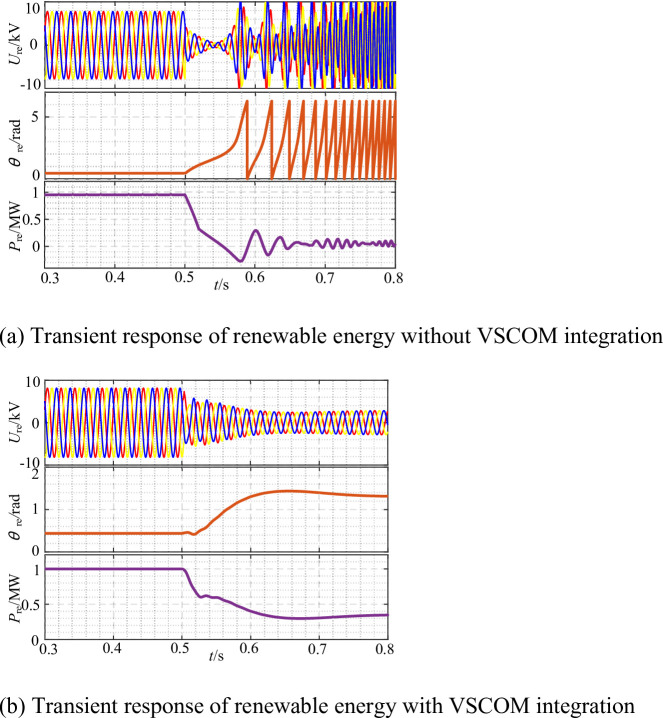
Influence of VSCOM on the transient stability of renewable energy units under SCR = 2.5. (**a**) Transient response of renewable energy without VSCOM integration, (**b**) Transient response of renewable energy with VSCOM integration.

To verify the improvement effect of VSCOM on the transient stability of the REGC under weak grid conditions, a simulation model was established with SCR = 2.5, and the REGC was operated at rated output. At t = 0.5 s, the grid voltage was reduced to 0.4 p.u. As shown in Fig. 13(a), without VSCOM integration, although a new system equilibrium exists after the fault under SCR = 2.5, the REGC operating with grid-following control lacks sufficient damping. Therefore, the PLL output phase of the REGC continuously increases after the grid fault, ultimately leading to transient instability. Figure 13(b) presents the simulation results with VSCOM integrated. After VSCOM connection, the dynamic response of the system is dominated by the VSCOM, which provides stronger damping. Therefore, the REGC maintains stable operation throughout the fault period. The simulation results demonstrate that VSCOM integration significantly enhances the transient synchronous stability of the REGC under weak grid conditions.

### Validation of the influence of VSCOM damping and inertia parameters on the transient synchronous stability of REGC

**Fig. 14:**
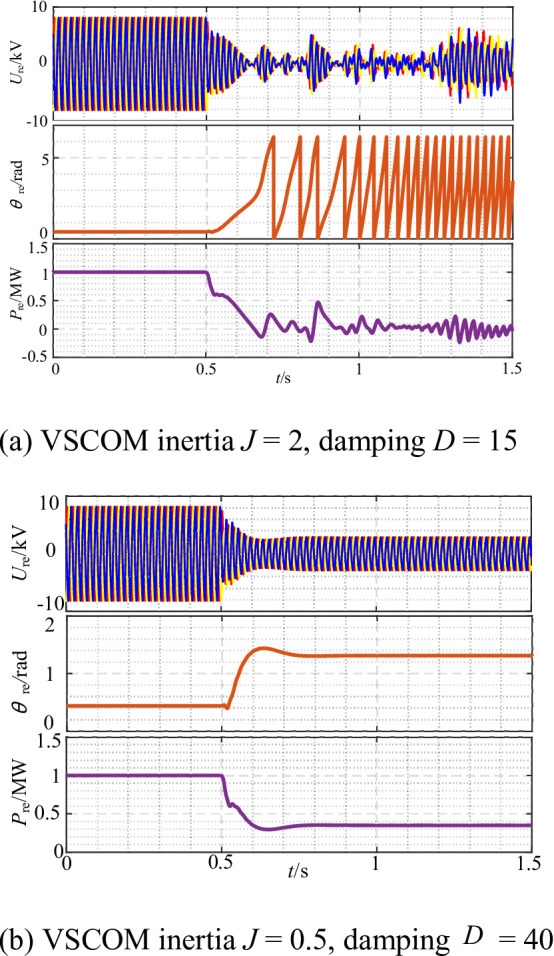
Output response of renewable energy units after VSCOM integration with different inertia and damping parameter settings. (**a**) VSCOM inertia J = 2, damping D = 15.

To assess the influence of VSCOM damping and inertia parameters on the transient synchronous stability of the REGC, the simulation model was configured with SCR = 2.5, and the grid voltage dropped to 0.4 p.u. at t = 0.5 s. The damping (*D*) and inertia (*J*) parameters of the VSCOM were varied for comparative simulations. The output response of the REGC under different parameter settings is shown in Fig. 14.

As shown in Fig. 14(a), with *D* = 15 and *J* = 2, the REGC experienced transient instability after the grid voltage sag. This instability results from excessive inertia combined with insufficient damping, which compromises system stability. In contrast, Fig. 14(b) demonstrates that increasing the damping coefficient to *D* = 40 and reducing the inertia to *J* = 0.5 allows the REGC to maintain stable operation after the voltage sag.

### Validation of the influence of VSCOM reactive power capacity on the transient synchronous stability of REGC

**Fig. 15:**
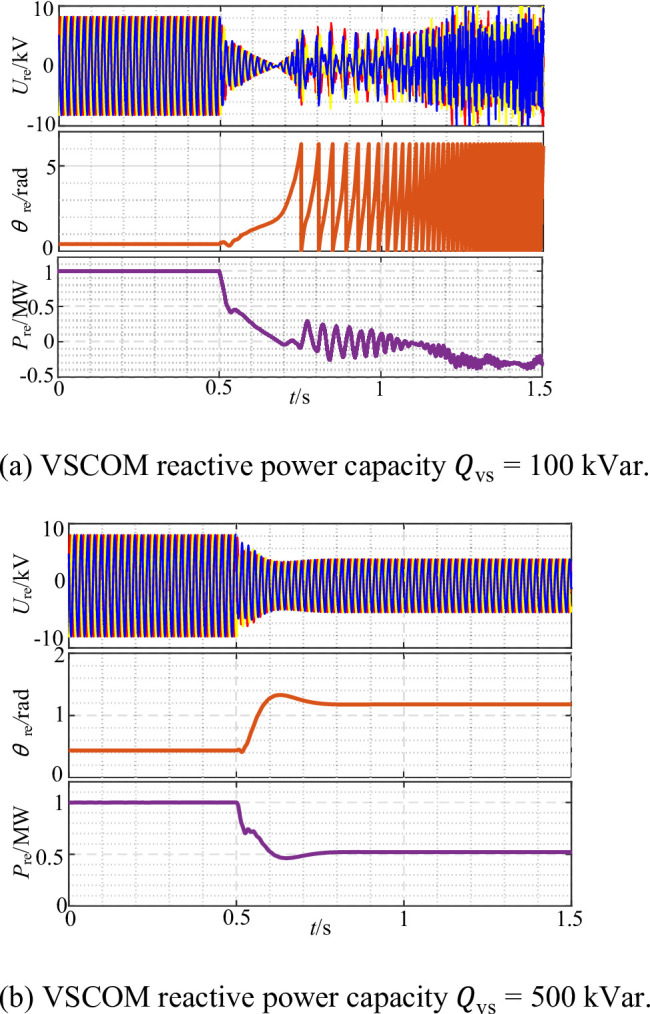
Output response of renewable energy units with VSCOM integration under different reactive power capacity settings. (**a**) VSCOM reactive power capacity Qvs = 100 kVar., (**b**) VSCOM reactive power capacity Qvs = 500 kVar.

To investigate the influence of VSCOM reactive power capacity on the transient synchronous stability of the REGC, simulations were conducted with SCR = 2.5, and the grid voltage dropped to 0.4 p.u. at *t* = 0.5 s. Comparative simulations were performed by varying the rated reactive power capacity of the VSCOM. The REGC output responses under different capacities are presented in Fig. 15.

When the VSCOM reactive power capacity was set to 100 kVar, as shown in Fig. 15(a), the REGC became transiently unstable following the voltage sag. This instability was due to insufficient reactive capacity of the VSCOM to provide the necessary voltage support to the REGC. As illustrated in Fig. 15(b), increasing the VSCOM reactive power capacity to 500 kVar enabled the REGC to maintain stable operation under the same voltage sag condition. The enhanced voltage support at the POC provided by the increased reactive capacity effectively improved system stability. These results confirm that increasing the VSCOM reactive power capacity is an effective measure for enhancing the transient synchronous stability of REGC systems.

## Conclusion

This study proposed a current-limiting strategy suitable for the VSCOM, analyzed the enhancement effect of VSCOM on the static stability limit of the REGC, and investigated the mechanism by which VSCOM affects the transient response of the REGC. Additionally, the influence of grid SCR, key VSCOM parameters, and reactive power capacity on the transient synchronous stability of the REGC was examined. The main findings and conclusions are as follows:

1. Based on the grid-forming voltage source characteristics of the VSCOM, a constant voltage current-limiting control method was proposed, ensuring effective voltage support from the VSCOM to the renewable energy plant during grid faults.
2. The voltage source characteristic of the VSCOM maintains a constant voltage at the POC and significantly improves the static stability limit of the REGC, particularly under weak grid conditions.
3. The synchronization loops of the VSCOM and REGC are coupled through their phase angles and the POC voltage. The degree of coupling depends on the electrical distance between the VSCOM and REGC. When the coupling is strong, the VSCOM dominates the transient characteristics of the REGC, which is beneficial for improving the transient stability of the REGC under weak grid conditions.
4. With VSCOM integration, reducing the *J*, increasing the *D*, and enhancing the reactive power compensation capacity further improve the transient synchronous stability of the REGC.

It should be noted that this study focuses on symmetrical faults, whereas actual power grids may experience more complex scenarios, such as asymmetric faults and frequency excursions following a fault. Investigating the performance and stability of the VSCOM-renewable energy system under these conditions constitutes a critical area for future research.

## Data Availability

The original contributions presented in the study are included in the article, further inquiries can be directed to the corresponding author.
